# Development of triazine non-nucleoside reverse transcriptase inhibitors for microbicidal applications

**DOI:** 10.1186/1742-4690-8-S2-P1

**Published:** 2011-10-03

**Authors:** Kevin K Ariën, Muthusamy Venkatraj, Johan Míchiels, Jurgen Joossens, PieterVan der Veken, Jan Heeres, Said Abdellati, Vicky Cuylaerts, Tania Crucitti, Paul Lewi, Koen Augustyns, Guido Vanham

**Affiliations:** 1 Department of Biomedical Sciences, Institute of Tropical Medicine, B-2000 Antwerp, Belgium; 2 Laboratory of Medicinal Chemistry, University of Antwerp, B-2000 Antwerp, Belgium; 3 Department of Clinical Sciences, Institute of Tropical Medicine, B-2000 Antwerp, Belgium

## Background

In search of antiretrovirals with microbicide potential, we have synthesized a library of non-nucleoside reverse transcriptase inhibitors (NNRTIs), encompassing 71 triazine analogues. We present data on the anti-HIV activity and toxicity using a broad armamentarium of *in vitro* assays and models.

## Materials and methods

In a primary screen, the anti-HIV activity against the laboratory strain Ba-L and against a primary subtype C isolate was determined in the TZM-bl cell line. Cellular toxicity on TZM-bl cells was evaluated using WST-1. Subsequently, a selection of 17 compounds was further evaluated for anti-HIV activity in different primary cells, including peripheral blood mononuclear cells, dendritic cells and CD4+ T lymphocytes. In addition, the activity against NNRTI-resistantviruses (V106A, Y181C, L100I/ K103N) was tested. The toxicity profile was further investigated using blood cells and epithelial cells originating from the female genital tract (FGT) and in a dual chamber assay modeling the FGT and underlying mucosae. Finally, toxicity towards vaginal flora (reference strains of *L.vaginalis*, *L. iners*, *L. jensenii*, *L. gasseri*, *L. crispatus*, A *vaginae*, *G. vaginalis*) was measured for the lead molecules UAMC00838 and UAMC01009. Dapivirine (TMC120) was used as a bench mark throughout the study.

## Results

In TZM-bl cells, most of the compounds were highly active against Ba-L and subtype C, with low nanomolar EC50 values slightly above or below the EC50 of dapivirine (2.0 nM). Similar nM activities were found in primary cells for a selection of 17 compounds. Interestingly, these compounds retained fairly good potency (EC50 values = 1-300 nm) against a resistant strain carrying the NNRTI-resistance mutations V106A or Y181C. However, potency was diminished (submicromolar and micromolar EC50 values) when tested against a mutant virus carrying L100I/K103N. Compounds UAMC00838 and UAMC01009 were identified as lead molecules based on their activity/toxicity profile and chemical structure.

These novel compounds showed similar or better toxicity profiles as the bench mark molecule dapivirine, in TZM-bl cells as well as in FGT epithelial cells and in a dual chamber system modeling the FGT. Finally, while no toxicity against vaginal lactobacilli was observed up to concentrations approx. 80,000 times above the EC50 of compound UAMC00838, the growth of G. *vaginalis* and A *vaginae* was inhibited at the highest compound concentration. Compound UAMC01009 showed additional low level toxicity against *L*. *iners* and high level toxicity against *L*. *crispatus.*

## Conclusions

We present data of highly active NNRTIs with a favorable toxicity profiles compared to the bench mark NNRTI dapivirine. Compound UAMC00838 does not affect the normal vaginal flora, but could inhibit G. *vaginalis* and A *vaginae*, which are associated with bacterial vaginosis, a risk factor for HIV acquisition. Ongoing studies on solubility and formulation will reveal their potential as intravaginal/-rectal microbicides.

